# Experiences of Ageism in mHealth App Usage Among Older Adults: Interview Study Among Older Adults Based on Extended Unified Theory of Acceptance and Use of Technology and Risks of Ageism Models

**DOI:** 10.2196/79457

**Published:** 2026-01-07

**Authors:** Jiayi Sun, Yawen Liu, Chengrui Zhang, Ying Xing, Wanqiong Zhou, Wei Luan

**Affiliations:** 1 Shuguang Clinical Medical College, Shanghai University of TCM, Shanghai, China; 2 Nursing Department, Shuguang Hospital Affiliated to Shanghai University of TCM, No. 528, Zhangheng Road, Pudong New Area, Shanghai, 201203, China, 86 021-20256016; 3 Shanghai Jiao Tong University School of Nursing, Shanghai, China

**Keywords:** mHealth apps, ageism, technology use, older adults, healthy aging

## Abstract

**Background:**

As the global aging population accelerates, mobile health (mHealth) apps have emerged as critical tools in the health management of older people. However, the promotion of mHealth apps has faced multiple obstacles, including insufficient technological adaptation to aging, digital resistance, and ageism. The impact of ageism on technology usage experiences among older adults is influenced by mechanisms such as stereotypes and biases. Notably, extant research has not adequately explored the subjective experiences of older adults in the context of mHealth app usage scenarios.

**Objectives:**

The present study was predicated on the extended unified theory of acceptance and use of technology model and the risks of ageism model to systematically explore and understand older adults’ ageism experiences in mHealth app usage. Our objectives were to provide a reference for optimizing age-friendly design and enhancing digital health management capabilities for older adults.

**Methods:**

This qualitative study utilized an interpretive phenomenological design and was conducted between February and April 2025. Purposive sampling was employed to select older adults with experience using mHealth apps in a Shanghai community for semistructured interviews. This study used Colaizzi’s phenomenological method to analyze and summarize older adults’ experiences and perceptions of ageism and to extract themes.

**Results:**

The study identified 3 core themes: (1) internalized age stereotypes, which manifest as technological uselessness and learning barriers; (2) anxiety and avoidance behaviors caused by stereotype threat; and (3) external unfair treatment (such as age-friendly design flaws and inadequate support systems), which inhibits usage. These experiences significantly impact older adults’ intention to use mHealth apps.

**Conclusions:**

Ageism profoundly affects the engagement of older adults with mHealth apps. It is advisable to execute systematic interventions to improve digital inclusion and health self-management capabilities, including strategies to challenge age stereotypes, optimize intergenerational support, refine age-friendly design, and establish strong social support networks.

## Introduction

### Background

The aging process of China’s population is accelerating and has reached an advanced stage of development. Recent statistics indicate that by the conclusion of 2024, China’s population aged 60 years and older will approximate 310 million, with those aged 65 years and older reaching around 220 million. These groups represent 22% and 15.6% of the total population, respectively [1]. As the aging process advances, the population of older internet users continues to expand. The 55th Statistical Report on China’s Internet Development indicates that the population of internet users aged 60 years and above has increased from 7.3 million in 2009 to 157.25 million in 2024 [2]. The internet has progressively emerged as a significant platform for older adults to obtain health information and services. Additionally, the incidence of chronic diseases in older adults has attained 75%, necessitating increasingly individualized and varied health care requirements [3]. This requires transcending conventional health care approaches to address the personalized health needs of the digital era [4]. The State Council of China has released a medium- to long-term plan (2017‐2025) on the prevention and treatment of chronic diseases, proposing the use of information technology to promote health management [5]. Mobile health (mHealth) apps enable remote diagnostics and personalized therapies [6], particularly aiding older adults by enhancing health care access and fostering health autonomy [7]. Nevertheless, older adults exhibit low awareness and utilization rates of mobile health care [8], attributable to inadequate age-appropriate design, technological resistance, and insufficient social support [8-10]. To resolve this issue, it is necessary to improve nationwide access to digital health services. This necessitates the systematic elimination of obstacles to digital health adoption among older adults through the refinement of technical standards, optimization of service systems, and enhancement of social support.

Notably, ageism has been demonstrated to intensify the digital divide [11,12]. The World Health Organization [13] defines ageism as stereotyping, prejudice, or discrimination based on actual age [14]. This includes self-directed ageism, which refers to negative internalized beliefs about aging [15], as well as benevolent forms, such as overprotection, and hostile forms, including neglect and judgment [16,17]. Ageism impacts older adults via 3 mechanisms: internalized stereotypes, avoidance strategies, and direct discrimination experiences [18]. These mechanisms undermine the psychological well-being of older adults and exert various negative impacts on their physical and mental health [19]. Research demonstrates that ageism markedly diminishes life satisfaction among older adults, intensifies social isolation, hinders chronic disease management outcomes, and elevates the risk of depression and cognitive decline [20]. Moreover, ageism may instigate apprehension regarding operational mistakes and reduce self-efficacy by leading older adults to internalize adverse stereotypes such as “techno-phobia.” This markedly diminishes their willingness to engage with mHealth programs, thereby hindering their capacity to access health resources through mHealth apps [21]. Previous research has mostly concentrated on quantitative analyses of influencing factors [22-24]; however, it has insufficiently addressed the subjective experiences of older adults. Research on mHealth apps has identified issues such as inadequate aging [25,26], yet it seldom explores the subjective psychological effects resulting from ageism.

### Objective

In order to investigate how older persons actually perceive ageism in the use of mHealth apps, this study takes an interpretive phenomenological method. It provides a reference and foundation for enhancing the age-friendly mHealth app design and creating an inclusive social support network, which will encourage older individuals’ digital integration and health management capabilities.

### Theoretical Framework

In 2003, Venkatesh et al [27] proposed the unified theory of acceptance and use of technology (UTAUT) model, which posits that user behavior in adopting technology is explained by four core dimensions: performance expectation (PE), effort expectation (EE), social influence (SI), and facilitation conditions (FC). The model demonstrated an explanatory power of 70%. It has been extensively used in research examining the adoption of new technologies among older adults [28]. This study examines privacy risks in mobile health care services by including a perceived risk (PR) dimension into the UTAUT model to create an enhanced framework. That improvement has been substantiated in the domain of medical technology [29,30].

The risks of ageism model (RAM) proposed by Swift et al explains the obstacles to positive aging through 3 pathways [18]: stereotype embodiment (negative labels imposed on older people by society), stereotype threat (self-doubt caused by older people internalizing negative labels), and being a target of ageism. The RAM model’s psychosocial mechanisms are not covered by the extended UTAUT model, although it can evaluate older adults’ desire to use mHealth apps due to its five dimensions: PEs, EEs, SI, enabling factors, and PR. The RAM model focuses on the manifestation of ageism and is not directly related to technology acceptance behavior.

**Figure 1.**
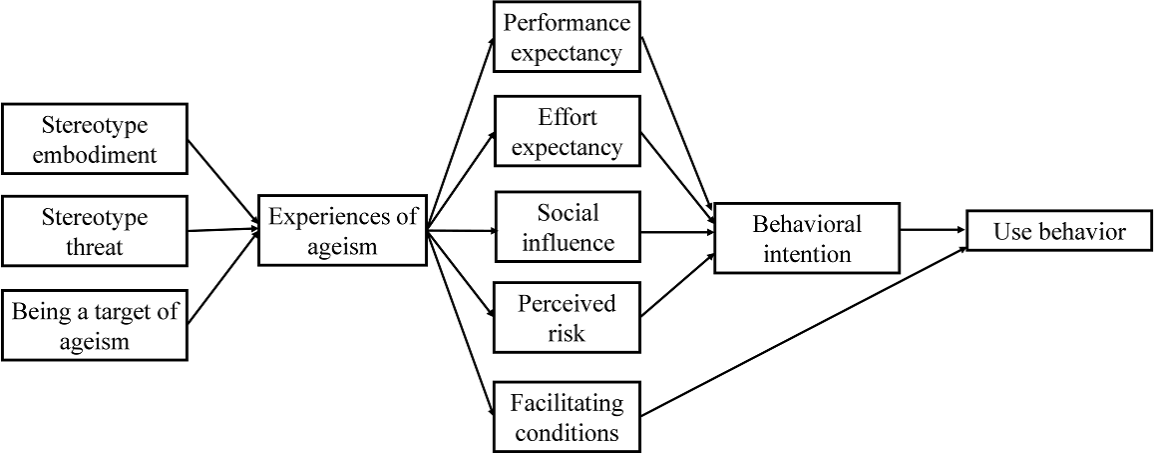
Extended unified theory of acceptance and use of technology and risks of ageism models.

The extended UTAUT model, primarily utilized in quantitative research, encompasses 5 core dimensions that systematically address essential factors influencing technology acceptance behavior, thereby offering a thorough theoretical framework for examining the digital health usage behavior of older adults. This study applies qualitative research to examine the influence of ageism on older adults’ perceptions and experiences within the UTAUT dimensions, aiming to reveal underlying mechanisms that quantitative research may not address. To establish a theoretical framework, as seen in Figure 1, this study combines the 2 models and maps the 3 routes of RAM to the 5 dimensions of UTAUT. This reveals how ageism affects the mechanism of interaction between the intention to use mHealth apps and behavior through these dimensions.

## Methods

### Design

We conducted face-to-face semistructured interviews from February to April 2025. This study employed a qualitative research design based on interpretive phenomenology. This approach was chosen to deeply explore the subjective experiences and psychological sentiments of older adults concerning ageism in their usage of mHealth apps from their own viewpoints. This study used Colaizzi’s method to understand individuals’ real experiences and experiences [31]. We followed the COREQ (Consolidated Criteria for Reporting Qualitative Research) reporting guideline to guarantee rigor and transparency.

### Participant Selection

We employed purposive sampling to select older adults in a community in Shanghai based on gender, age, and mHealth app usage experience. The selection criteria for interviewees were as follows: (1) individuals aged 60 years or older, who have resided in the community for a minimum of 5 years (or at least 10 mo/y); (2) individuals with normal cognitive function, adequate physical strength to participate in the interview, and the ability to communicate in Mandarin; (3) regular use of mHealth apps for at least 3 months; and (4) voluntary consent obtained through the signing of an informed consent form. Exclusion criteria included the presence of mental illness or cognitive impairment, severe hearing or language impairment, and withdrawal from the study. We adopted the principle of maximum differentiation sampling, with sample size determined by information saturation (ie, no new themes appeared in the interview content).

Eligible interviewees were recruited through community hospital nurses. The study conducted face-to-face semistructured interviews from February to April 2025, with the interview outline designed based on the RAM and UTAUT theoretical frameworks. The research design emphasized ethical approval to ensure the protection of participants’ privacy.

### Ethical Considerations

This study was approved by the hospital ethics committee (RA-2021‐465). All participants were informed that their participation was voluntary and anonymous and that no adverse consequences would result from the interview. All participants signed written informed consent forms after being informed of the study’s purpose, procedures, risks, and benefits, and received guidance on mHealth apps usage as compensation. We refer to all interviewees with a number (N) and a letter to ensure their anonymity.

### Setting

To facilitate comprehension among older adults regarding the study, information leaflets were handed out, and interviews were conducted in soundproof rooms to ensure privacy and minimize interference from unrelated individuals. The interviewer initially articulated the study’s purpose and significance to the interviewee, ensuring that their personal information would remain confidential. Following that, after establishing trust through a 30-minute warm-up, the interviewer conducted a semistructured interview lasting 25 to 35 minutes, employing the “dual recording method” (audio recording along with written notes). The interviewer dynamically adjusted the questioning strategy and documented nonverbal cues, such as facial expressions and body language, using information saturation as the criterion for termination. The interviewer (first author) transcribed the recorded interviews verbatim within 24 hours and invited the interviewees to verify the transcripts. Data were encrypted and stored, accessible only by the project team within an ethical framework, in full compliance with the Declaration of Helsinki requirements.

### Data Collection

After obtaining approval from the community hospital, we contacted the hospital staff to determine the visit time. Before inviting eligible residents to sign the informed consents, the researcher (first author) explained the study to them. After signing the informed consents, data were collected through semistructured face-to-face interviews and observations. The first author conducted individual semistructured face-to-face interviews from February to April 2025.

Following the identification of participants, researchers engaged with them to observe their daily utilization of mHealth apps, including the types of apps and their level of proficiency in usage. Using the extended UTAUT and RAM theoretical framework, we employed an interpretive phenomenological approach to develop semistructured interview outline. Following 2 rounds of Delphi expert consultation (n=5) and 3 discussions among the research team, along with practical feedback from 2 preliminary interviews (n=2), we developed a semistructured interview outline consisting of 6 theoretical dimensions: (1) EE: Which mHealth apps have you used? Can you give us a brief overview of your learning and usage experience? (2) PE: What is your attitude toward mHealth apps? Have they met your expectations? (3) SI: What is the attitude of your family, friends, or health care providers toward your usage of mHealth apps, and how does their attitude influence your usage? (4) FC and PR: What factors do you think influence your usage of mHealth apps? (5) In your opinion, what kind of mHealth app would be most suitable for you and your peers to use? (6) What kind of help would you like to receive when using mHealth apps? The research process strictly followed qualitative research standards. All interview questions were optimized for readability (Flesch-Kincaid index≤6.0) and cognitive adaptability testing to ensure that they were understandable to older interviewees.

Probing questions within the interview allowed participants to raise unexpected issues and provided flexibility to follow-up on these issues. By asking follow-up questions based on the answers to previous questions, interviewees were encouraged to freely share their experiences. The interview guide was used to ensure that all topics were covered.

### Data Analysis

This study employed a systematic qualitative data analysis approach: initially, interview data was anonymized and assigned identification codes, while data collection and analysis occurred concurrently. Transcription was finalized within 24 hours post-interviews, with accuracy confirmed by a research team member and observational notes incorporated as supplementary data. The Colaizzi’s method [31] was employed alongside NVivo 14.0 software to facilitate the analysis, resulting in a thematic map established through a 3-tier quality control process involving analysis by principal investigators, expert supervision, and review by the research team. Disputed content was addressed through consensus meetings to ensure the research’s rigor and the interpretation’s reliability.

### Rigor

The content of the interview outline was determined through literature review and theoretical framework, and semistructured interviews were conducted with the target group in advance (not included in the study) to ensure that the interview outline was rigorous and easy to understand. The interviews were carried out by master’s degree nursing students trained in qualitative research methodologies. To ensure the reliability of the collected data, we employed a combination of prolonged exposure, comprehensive analysis, diverse information sources, and various data collection methods, including interviews, field notes, and member checking by colleagues and participants. In the data analysis, efforts were made to incorporate the interviewees’ emotions while minimizing the influence of the researchers’ preconceived notions.

## Results

### Overview of Data and Analysis

**Figure 2.**
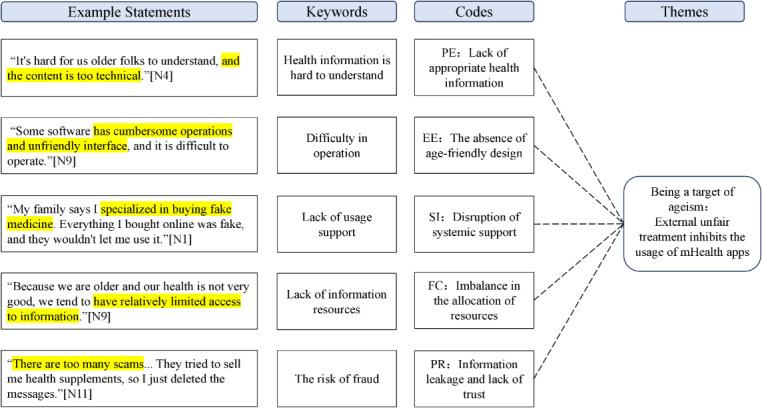
Process of thematic analysis (theme 3). EE: effort expectation; FC: facilitation condition; PE: performance expectation; PR: perceived risk; SI: social influence.

The study achieved data saturation after conducting interviews with 12 participants, at which point no new codes emerged, leading to the termination of the interviews. A total of 12 older adults were included in the study. The total duration of the interviews was approximately 370 minutes, with the transcripts of the relevant themes totaling around 50,000 words. Table 1 presents the general information of the 12 interviewees. The analysis process is shown in Figure 2, which only showed the analysis process of Theme 3.

**Table 1.** General information of the interviewees (n=12).

Code	Gender	Age, y	Monthly household income^a^	Education	Living arrangement	Marital status	Health insurance	Self-reported health status
N1	Female	66	3000‐5000 (US $420-700)	Middle school	Three-generation co-residence	Married	URBMI^b^	Excellent
N2	Female	67	3000‐5000 (US $420-700)	Middle school	Living with spouse	Married	UEBMI	Excellent
N3	Female	77	5000‐10,000 (US $700-1400)	High school	Living alone	Widowed	UEBMI^c^	Good
N4	Male	73	5000‐10,000 (US $700-1400)	Middle school	Living with spouse	Married	URBMI	Fair
N5	Male	75	1000‐3000 (US $140-420)	High school	Living with spouse	Married	URBMI	Good
N6	Female	69	5000‐10,000 (US $700-1400)	High school	Living alone	Divorced	URBMI	Fair
N7	Female	65	5000‐10,000 (US $700-1400)	High school	Living with spouse	Married	URBMI	Good
N8	Male	72	3000‐5000 (US $420-700)	High school	Living with children	Married	NCMS^d^	Fair
N9	Female	74	3000‐5000 (US $420-700)	High school	Living with spouse	Married	UEBMI	Good
N10	Male	63	1000‐3000 (US $140-420)	Middle school	Three-generation co-residence	Married	NCMS	Fair
N11	Male	75	5000‐10,000 (US $700-1400)	High school	Living with spouse	Married	URBMI	Fair
N12	Female	66	5000‐10,000 (US $700-1400)	Middle school	Living with spouse	Married	UEBMI	Good

a A currency exchange rate of CNY 1 = US$ 0.14 (as of December 2025) is applicable for converting monthly household income.

b URBMI: Urban Resident Basic Medical Insurance.

c UEBMI: Urban Employees Basic Medical Insurance.

d NCMS: New Cooperative Medical Scheme.

### Theme 1: Stereotype Embodiment

#### PE: Age Labeling Weakens the Perceived Value of mHealth Apps

The internalization of age-related stereotypes caused participants to associate advanced age with a decrease in technological skills, resulting in a self-perception of “technological uselessness.” The reduction in self-efficacy significantly impaired their acknowledgment of the potential health management advantages provided by mHealth apps. As N11 articulated:

*I’m 75 years old. Why should I learn anything new? I’m getting old. These mHealth apps won’t help much.*

It is worth noting that some interviewees actively use technology to compensate for age-related cognitive decline, reflecting compensatory strategies in individual behavior under the threat of stereotypes.

*If there is something wrong with my body, I will look for answers.*

[N9]

*The doctor also talked about it (health-related knowledge), but we don’t have very excellent recollections, so we can’t remember anything. That’s why we look for information online on our own.*

[N10]

#### EE: Magnification Technology Usage Barriers Attributed to Age

Participants frequently ascribe operational challenges to the unavoidable cognitive and physical decline linked to aging, rather than to design deficiencies or insufficient guidance, thus exacerbating perceived obstacles to usage. N12 stated:

*It’s best if it’s simple (in terms of operation and functions). We (elderly people) sometimes don’t really understand (complex) programs.*

N11’s attribution to educational background: “We have a relatively low level of education, so we don't really know how to use many of the functions, and it’s quite difficult to learn,” forming self-imposed barriers to technical learning. This cognitive pattern of attributing operational failures to age is essentially a concrete manifestation of implicit age stereotypes in the field of technology.

#### SI: Family Environment Reinforces Negative Expectations About Technology

External stereotypes are internalized as self-perceptions through significant individuals, particularly family members. Respondents reported that specific attitudes held by family members—such as the belief that “older adults are easily misled” (N10) and the idea that basic mobile phones are adequate for seniors (N8)—contributed to their negative self-perceptions about technological abilities. This indicates that the perpetuation of negative stereotypes within the social milieu substantially impedes the inclination to engage with technology.

*My family told me not to believe what I read online because it’s all scams aimed at old people, and they’re worried that I’ll get scammed.*

[N10]

*My kids said that basic phones for seniors are fine, so why do I need a smartphone?*

[N8]

#### FC: Self-Imposed Constraints Hinder the Utilization of External Resources

Internalized stereotypes are evident in 2 specific behavioral patterns: intentional avoidance of acquiring new technologies and an overdependence on intergenerational support. The self-identification as “unlearnable” served as a significant obstacle, hindering the effective conversion of available external support resources into realized technical skills. As a result, a disconnect arose between the availability of facilitating conditions and their actual behavioral application, thereby constraining the practical adoption of mHealth apps.

*We’re not like young people; we don’t want to learn new functions; these functions are enough for us.*

[N12]

*I’m lazy. I won’t learn how to use (mHealth apps) unless I have to. My kids will help us if I need it.*

[N5]

### Theme 2: Stereotype Threat

#### PE: Avoidance of Perceived Value of mHealth Apps

Internalized age stereotypes diminish older individuals’ confidence in the health advantages of technology, thereby creating a cycle of self-fulfilling prophecy. N12 said,

*I can’t find the answers I want (in the software) … I like going to the hospital to visit a doctor more because I trust what they say more.*

This preference indicates a strategic avoidance of digital health information, motivated by a concern that unsuccessful attempts may reinforce the stereotype that older individuals are unable to effectively use technology. Meanwhile, N10’s prudent disposition (“My mind works slowly, so I’m not very willing to try new things unless they’re truly useful”) illustrated the inhibition of perceived technological efficacy induced by stereotype threat, stemming from the allocation of working memory resources to anxiety.

#### EE: Psychological Attributions for Avoiding Technical Learning

Older adults attributed operational challenges to the inevitability of aging, creating a cognitive pattern of technical inertia. The assertions “I’m getting older and don’t want to learn new things” in N7 and “I’m not familiar with it (mHealth apps) and am too lazy to learn; my son will assist me” in N4 suggest that age-related labels generate negative psychological expectations and significantly reduce the motivation to develop technological skills. This attribution corresponds with the behavioral pattern of stereotype threat characterized by low expectations and low investment, leading to additional impairment of executive processes, such as cognitive flexibility.

#### SI: Usage Avoidance Under Interpersonal Pressure

External stereotypes were internalized as self-restrictive ideas through intergenerational exchanges. N8 expressed that his children’s belief that “the elderly are easily deceived” made him feel “afraid to look, lest my children get into trouble,” while N10 stopped asking questions after encountering his children’s “impatient” attitude, illustrating how adverse feedback within the familial context reinforced avoidance behavior. This social pressure may be viewed as a manifestation of “group identity threat,” leading older adults to preserve interpersonal harmony by refraining from technological involvement.

#### PR: Safety Concerns Intensify the Crisis of Confidence

Technical risk issues combined with traditional age biases resulted in a dual barrier to trust. The primary concerns for older adults using mHealth apps were the security of personal information and the protection of property. N3’s awareness of concealed charges (“they charge you after asking a few questions”), N2’s apprehension and concern regarding potential fraud (“you will be scammed if you click on it”), and N12’s doubt regarding the accuracy of information (“it’s a mix of truth and falsehood, so it’s better to go to the hospital”) illustrated the spillover effect of stereotype threat; anxiety consistently undermined trust in technology, even when detached from specific contexts.

### Theme 3: Being a Target of Ageism

#### PE: Lack of Appropriate Health Information

Systematically disregarding the cognitive traits of older adults resulted in ineffective health content. N3 indicated that “the software content (in the software) is too difficult to understand… too technical and hard to remember,” while N4 complained that “the content is too technical.” These perceptions indicate a failure in information design to accommodate age-related changes in cognitive processing, such as declines in working memory capacity, thereby reducing the perceived usefulness and accessibility of the information provided. N5 revealed deceptive advertising (“the recommended products claim to lower blood sugar, but there is no scientific basis for this”), which decreased trust in technology and highlighted the exploitation of older adults for profit.

#### EE: The Absence of Age-Friendly Design

There were considerable age-related deficiencies in interface interaction. N4 emphasized the lack of physiological adaptation, stating that “There is no version specifically designed for the elderly... the font should be enlarged.” N8 emphasized the necessity for multimodal requirements, asserting that “the voice version should be directly audible.” N9 criticized the “cumbersome operations and unfriendly interface,” specifically highlighting the design issue of prioritizing “technology-centric” over “user-centric” in product development. The lack of age-appropriate design increased the learning burden for older adults and reduced the user experience.

#### SI: Disruption of Systemic Support

The intergenerational influence within families significantly impacted technology usage. N1 was designated as “specialized in buying fake medicine” and prohibited from using mHealth apps, while N4 encountered limitations due to his children’s concerns regarding possible financial exploitation, demonstrating that protective measures resulted in technological deprivation.

The health care system offered minimal professional support. N10 noted, “The medical staff didn’t mention it and told me to go to the hospital,” whereas N1 stated, “Sometimes when I ask them, they get a little impatient.”

#### FC: Imbalance in the Allocation of Resources

Public health education resources were insufficiently integrated into digital platforms. The lack of resources intensified the technological marginalization of older adults. Some older adults exhibited confidence in official information sources and a desire to gain knowledge.

*There are no such platforms available on a regular basis, so I don’t know what software is available or which software is suitable for us. Apart from what the nurses at the hospital recommend, I don’t know anything else.*

[N8]

*Because we are older and our health is not very good, we tend to have relatively limited access to information.*

[N9]

#### PR: Information Leakage and Lack of Trust

Older adults often mentioned challenges associated with breaches of personal information, excessive promotional communications, and misleading marketing practices from unregistered medical institutions.

*I don’t know how they got my personal information. Many “doctors” send me text messages.*

[N10]

They recommend products that claim to cure diabetes, but that’s a scam… We also try to verify the authenticity of such information.

[N8]

There are too many scams… They tried to sell me health supplements, so I just deleted the messages.

[N11]

## Discussion

### Principal Findings

This study examined the ageism encountered by older adults in the community while using mHealth apps and the mechanisms through which it impacts them. The primary findings indicate that ageism obstructs the adoption and utilization of mHealth apps via 3 principal pathways of the RAM model: Stereotype internalization in older adults leads to the attribution of operational difficulties to aging, resulting in a self-perception of technological incompetence. This perception diminishes their acknowledgment of the benefits of mHealth and establishes a challenging learning threshold to surpass. Stereotype threat induces anxiety avoidance, characterized by fears of privacy breaches, worries regarding operational failures, and excessive protection or negative feedback from family members. These factors collectively result in the active avoidance of technology exploration and usage. External unfair treatment, evident in age-inappropriate design flaws in apps, including complex interfaces, limited information availability, and insufficient support systems, creates barriers to usage and diminishes the perceived value of these apps. Research indicates that ageism operates at various levels, including individual cognition, social interaction, and the technological environment, creating substantial barriers that impede older adults’ integration into digital health.

### Reconstructing Digital Health Cognition in Older Adults: A Dual-Pathway Intervention to Enhance mHealth App Usage Effectiveness

Older adults typically internalize the stereotype that “age dictates technological ability.” This belief leads to attributing operational failures to age, avoiding the learning of new technologies, and concerns about inconveniencing others. Consequently, this significantly diminishes their willingness to use mHealth apps and undermines their sense of self-efficacy [32]. This cognitive pattern establishes obstacles in 3 dimensions—PE (diminished perceived health value), EE (reduced learning motivation), and FC (increased perceived learning costs)—illustrating the self-fulfilling prophecy of stereotype threat within the RAM model [33-35], thereby compromising well-being and impacting physical health [36]. Interventions should implement a dual approach to address this phenomenon. Positive narratives should be employed to highlight success stories among peers, such as in chronic disease management, underscoring that proficiency with technology is contingent upon practice rather than age, while minimizing the notion of “technological disadvantage.” Meanwhile, an educational approach characterized by “low threshold+high feedback” should be adopted. This involves deconstructing essential functions, such as blood pressure monitoring and medication reminders, while offering voice navigation and immediate feedback to build successful experiences, improve self-efficacy, and encourage proactive health behaviors [37].

### From Substitution to Empowerment: Developing a Novel Paradigm for Digital Health Support for Older Adults

Research indicates that benevolent ageism, characterized by overprotective family members and insufficient support from medical personnel, exacerbates older individuals’ perceptions of incompetence [16]. The original intention may be to protect older adults; however, it implicitly stereotypes them as deficient in information, judgment, and technical skills, thereby diminishing their willingness to utilize mHealth apps [17,18]. This discrimination establishes a vicious cycle of “external rejection-self-rejection-behavioral withdrawal” through the “being a target of ageism” path in the RAM model and the “social influence” dimension in the UTAUT model. Studies indicate that the advice and trust that kids give notably affect older adults’ willingness to adopt technology, while positive interactions between generations may improve perceptions of aging [38], Studies indicate that the advice and trust that kids give notably affect older adults’ willingness to adopt technology, while positive interactions between generations may improve perceptions of aging [39]. Digital reverse mentoring should be promoted to enhance older adults’ information literacy, while improving medical staff’s negative perceptions of older adults [40]. The objective is to shift the support framework from “substitution” to “empowerment and accompaniment,” motivating older adults to assume control over device operation, acknowledging their advancements promptly, eliminating their self-perception as “technologically disadvantaged,” and enhancing their sense of worth as participants to digital health.

### Eliminating Invisible Technological Barriers: A Dual-Path Approach to Aging-Friendly Design and Risk Prevention in mHealth Apps

Research indicates that only 40% of mHealth apps incorporate older adults in the design process [41], and challenges such as intricate interfaces, superfluous operations, and insufficient information availability highlight invisible ageism at the technology level. Analysis based on the UTAUT model: in the EE dimension, design deficiencies such as small font sizes and poor-quality push notifications elevate learning costs [42]; in the PE dimension, an overabundance of technical terms and insufficient personalized guidance diminish perceived utility [43]; in the PR dimension, apprehensions regarding privacy breaches and misleading advertisements undermine usage intentions [44,45], all of which exacerbate older adults’ self-perceptions of technological discrimination [41,46]. We advocate adopting a dual-faceted strategy: in terms of technology, implement age-appropriate features such as adjustable font sizes and voice navigation [47], create specialized and accessible health information [48], and engage older adults in the initial design stages to mitigate stereotypes [41]. In terms of risk prevention and control, establish data protection methods to eliminate hazardous information [49], while strengthening governmental regulation to create a trustworthy environment. By systematically optimizing design and management processes, obstacles to utilization by older adults can be efficiently reduced, consequently enhancing digital health inclusivity.

### Multistakeholder Collaborative Empowerment: Constructing a Social Support System for Older Adults' Digital Health

Findings indicate that older adults frequently avoid engaging with mHealth apps due to a lack of informational resources and prevailing stereotypes (eg, “incapable of learning,” “poor judgment”) [50], which results in a diminished perception of resource support in the FC dimension. A multitiered support network is essential: medical institutions should incorporate mHealth guidance into health management services, and medical staff must acquire aging-friendly instructional skills to establish trust through recommendations from authoritative institutions. Research indicates that 55.5% of older internet users have encountered online risks [51], while also demonstrating significant vigilance [52]. Governments need to encourage platforms to enhance the digital environment, optimize recommendation algorithms, and implement antifraud training to bolster information discrimination capabilities. Through the combination of professional support from the medical system and social risk prevention and control, it is feasible to address older adults’ demand for authoritative information while mitigating digital risks. This approach enhances the perceived benefits of mHealth apps and promotes healthy aging.

This study systematically elucidates the psychosocial mechanisms by which age discrimination affects older adults’ adoption of mHealth apps, integrating and extending the UTAUT and RAM models. This study extends the application of the UTAUT model, confirming its efficacy and potential as a qualitative research framework for examining the subjective experiences of older adults. The integration of the RAM model with UTAUT elucidates the impact of ageism’s psychosocial mechanisms on critical aspects of technology acceptance, providing fresh insights into the digital health barriers faced by older adults. These findings have practical implications for improving the delivery of digital health services in the communities and hospitals of Shanghai. Community health promotion activities must challenge stereotypes regarding older adults, enhancing their confidence through peer modeling and prompt feedback. Second, families and health care institutions ought to transition from “substitute operation” to “empowering accompaniment,” thereby reducing psychological barriers through enhanced communication and initial guidance. Ultimately, the optimization of technology and services should be closely aligned with distinct user requirements, including interface complexity, information credibility, and the availability of continuous support.

### Limitations

First, the sample size of this qualitative study is relatively small. Although small, the sample has reached theoretical saturation, meaning that adding more participants is unlikely to yield new insights. The current sample is drawn exclusively from a single community in Shanghai, which may introduce geographical and cultural biases that limit the generalizability of the findings. Second, despite using purposive sampling, selection bias may still be present, as participants who are more positive or more persistent in their usage of mHealth apps are more likely to participate. To mitigate this effect, we deliberately recruited participants who reported lower levels of mHealth apps usage. Additionally, the study was limited to older adults living in the community and did not include older adults in institutions such as nursing homes, which may have overlooked the experiences of ageism among more vulnerable groups. Therefore, future studies should include a broader population for comparative analysis to gain a more comprehensive understanding of the experiences of ageism in the use of mHealth apps among different groups.

In addition, this study used qualitative research methods, which are inherently subjective. To improve the scientific rigor of our research, we referenced qualitative research quality standards and focused on the following areas: researchers engaged in all interviews, recordings, and transcription processes to establish trust with participants, thereby enhancing credibility. Purposeful sampling was utilized to enhance the transferability of findings, accompanied by comprehensive descriptions of the sample characteristics. To ensure dependability, all interviews were audio-recorded, and comprehensive interview notes, transcripts, and research reflection journals were preserved. To ensure confirmability, 2 researchers performed independent data analyses, sought third-party consensus in cases of discrepancies, and provided organized data to research participants for verification. Although we used rigorous qualitative analysis to minimize bias, incorporating quantitative methods could provide more objective data, thereby improving the credibility and scientific rigor of the results. Future studies should adopt a mixed-method approach, combining qualitative and quantitative data to gain a more comprehensive understanding, provide objective indicators to enhance the reliability of the research results, and quantify the impact of ageism on the usage of mHealth apps. Finally, the duration of this study may limit our ability to track long-term changes in older adults’ experiences of ageism. Future studies should include long-term follow-up to assess the temporal changes in ageism experiences and technology acceptance behavior, thereby providing more comprehensive insights and practical guidance for intervention design.

### Conclusions

In this study, based on the extended UTAUT and RAM models, we identified 3 significant issues related to ageism in the usage of mHealth apps among older adults in the community: internalized stereotypes, benevolent ageism, and aging-friendly design flaws. We propose intervention strategies throughout 4 dimensions: psychological cognition, support systems, technological optimization, and social networks. This research is confined to a singular community sample located in Shanghai. Future studies should enhance the geographical and cultural diversity of the sample, implement longitudinal tracking and quantitative methods, and perform comprehensive research on the causal relationship between ageism and mHealth apps usage behavior to establish a foundation for creating an inclusive digital health environment.

## Abbreviations

COREQ: Consolidated Criteria for Reporting Qualitative Research
EE: effort expectation
FC: facilitation conditions
mHealth: mobile health
PE: performance expectation
PR: perceived risk
RAM: risks of ageism model
SI: social influence
UTAUT: unified theory of acceptance and use of technology
